# Entropy Production and Its Application to the Coupled Nonequilibrium Processes of ATP Synthesis

**DOI:** 10.3390/e21080746

**Published:** 2019-07-30

**Authors:** Sunil Nath

**Affiliations:** Department of Biochemical Engineering and Biotechnology, Indian Institute of Technology Delhi, Hauz Khas, New Delhi 110016, India; sunath@iitd.ac.in or

**Keywords:** free energy dissipation in oxidative phosphorylation (OX PHOS), ATP synthesis by F_1_F_O_-ATP synthase, irreversible thermodynamics/nonequilibrium thermodynamics of biological processes, optimization of biological energy converters, variational principle, Mitchell’s chemiosmotic theory, Nath’s torsional mechanism of energy transduction and ATP synthesis, Nath’s two-ion theory of biological energy coupling, succinate as feedback signal and anion uncouplers of OX PHOS, biothermokinetics

## Abstract

Starting from the universal concept of entropy production, a large number of new results are obtained and a wealth of novel thermodynamic, kinetic, and molecular mechanistic insights are provided into the coupling of oxidation and ATP synthesis in the vital process of oxidative phosphorylation (OX PHOS). The total dissipation, Φ, in OX PHOS with succinate as respiratory substrate is quantified from measurements, and the partitioning of Φ into the elementary components of ATP synthesis, leak, slip, and other losses is evaluated for the first time. The thermodynamic efficiency, η, of the coupled process is calculated from the data on Φ and shown to agree well with linear nonequilibrium thermodynamic calculations. Equations for the P/O ratio based on total oxygen consumed and extra oxygen consumed are derived from first principles and the source of basal (state 4) mitochondrial respiration is postulated from molecular mechanistic considerations based on Nath’s two-ion theory of energy coupling within the torsional mechanism of energy transduction and ATP synthesis. The degree of coupling, q, between oxidation and ATP synthesis is determined from the experimental data and the irreversible thermodynamics analysis. The optimality of biological free energy converters is explored in considerable detail based on (i) the standard biothermodynamic approach, and (ii) a new biothermokinetic approach developed in this work, and an effective solution that is shown to arise from consideration of the molecular aspects in Nath’s theory is formulated. New experimental data in state 4 with uncouplers and redox inhibitors of OX PHOS and on respiratory control in the physiological state 3 with ADP and uncouplers are presented. These experimental observations are shown to be incompatible with Mitchell’s chemiosmotic theory. A novel scheme of coupling based on Nath’s two-ion theory of energy coupling within the torsional mechanism is proposed and shown to explain the data and also pass the test of consistency with the thermodynamics, taking us beyond the chemiosmotic theory. It is concluded that, twenty years since its first proposal, Nath’s torsional mechanism of energy transduction and ATP synthesis is now well poised to catalyze the progress of experimental and theoretical research in this interdisciplinary field.

## 1. Introduction

Nonequilibrium thermodynamics has proven to be a general and useful formalism for the description of coupled processes in physics, chemistry, biology, and engineering [1,2,3,4,5,6,7]. It has been successfully used to model both coupled chemical reactions as well as coupled transport of species in a variety of contexts [1,8,9,10,11]. Recently, important biomedical problems have also been treated within this framework [12].

In particular, the coupled chemical reactions culminating in the synthesis of adenosine triphosphate (ATP) by oxidative phosphorylation (OX PHOS) have been the focus of key studies in biothermodynamics [2,13,14]. The concept of the degree of coupling between the exergonic and endergonic reactions has been central to a quantitative description of the coupled nonequilibrium process [1,2,4,5,6,13].

Currently, most textbooks of biochemistry use Mitchell’s chemiosmotic theory for interpretation of ATP synthesis mechanism and thermodynamics [15,16]. However, it is an old theory, dating back to 1961 when precious little was known about this complex subject [17]. Hence alternative mechanisms that offer important new information [18,19,20] and take into account the many advances in the field during the past several decades should now be considered seriously.

The discovery of inter-subunit rotation in F_1_-ATPase by direct visualization using epifluorescence microscopy techniques [21], and the presence of symmetry mismatch between F_O_ and F_1_ in ATP synthase [22] necessitated the formulation of a new molecular mechanism of energy coupling in ATP synthase [23,24,25,26]. The mechanism, named Nath’s torsional mechanism of energy transduction and ATP synthesis by other authors, has been further embellished and woven into a consistent theory that integrates all the available information in the field [9,10,11,18,19,20,23,24,25,26,27,28,29]. Various aspects of the theory can also be understood by gleaning from the commentary articles, book extracts, and material written on it by other scientists [30,31,32,33,34,35,36,37,38,39,40,41]. Twenty years after its first proposal [23], it is interesting to understand how much its explanatory power can be stretched and to determine how well it responds to systematic experimental probing and tests of thermodynamic consistency.

In spite of the considerable research activity summarized above, there is no published work on OX PHOS that utilizes the universal concept of entropy production as a starting point and goes on to develop from first principles the key thermodynamic, thermokinetic, and mechanistic aspects of the subject. It would also be instructive to explore the new insights Nath’s theory can offer in such an endeavor, which may additionally serve as a litmus test for the theory’s explanatory power and its ability to integrate the available experimental information. The exercise and the results obtained in this invited paper for the Special Issue, “Entropy Production and its Applications: From Cosmology to Biology” would also be of scientific interest and relevance to the readership of *Entropy*.

The paper is organized as follows. Section 2 quantifies from experimental data the rate of entropy production in OX PHOS and its parsing into its various elementary reaction and transport steps. In Section 3, the thermodynamic efficiency of the coupled nonequilibrium process is calculated from knowledge of the rate of entropy production. This is tested by a linear nonequilibrium thermodynamic analysis of ATP synthesis in Section 4. In Section 5 and Section 6, the degree of coupling, q, between oxidation and ATP synthesis, and other kinetic parameters arecalculated from the data using nonequilibrium thermodynamics.

The optimality of biological free energy converters is explored in fairly minute detail in Section 7. A biothermodynamic approach is analyzed in Section 7.1, and a novel *biothermokinetic* approach based on Nath’s two-ion theory of energy coupling within the torsional mechanism is developed in Section 7.2. This paves the way for establishing molecular mechanisms that go beyond Mitchell’s chemiosmotic theory (Section 8) based on the new experimental data. A mechanistic scheme for coupling based on Nath’s theory is presented and its consistency with the experimental data is tested in Section 8.1 and Section 8.2.

## 2. Entropy Production During Redox-Coupled ATP Synthesis

The thermodynamic fluxes $J_{P},\;J_{O}$ as a function of the thermodynamic forces (affinities) $A_{P},\;A_{O}$ have been experimentally determined for the nonequilibrium processes of redox-coupled ATP synthesis in rat liver mitochondria with succinate as respiratory substrate [10,11]. In particular, these papers sought to quantify the state 3 to state 4 transition in mitochondrial oxidative phosphorylation. In this work the total rate of entropy production, or more exactly, since the system is isothermal, the temperature times the total rate of entropy production which measures the rate of free energy dissipation, Φ, and the partitioning of Φ into its elementary component reactions or transport steps has been calculated. These components include the free energy dissipation for the actual ATP synthesis reaction through the F_1_F_O_-ATP synthase, and the free energy dissipation due to mitochondrial membrane leaks, pump slips, and other losses such as those arising from the active transport of ions against their concentration gradients. In terms of equations,

(1) $$\Phi_{Total}=\underset{i}{\sum }J_{i}A_{i}=\Phi_{ATP\;synthesis}+\Phi_{leak}+\Phi_{slip+other\;losses}$$

**Figure 1:**
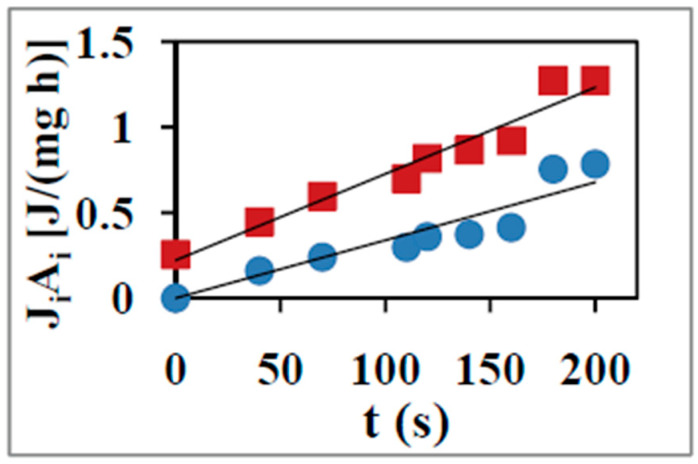
$J_{i}A_{i}$ (where i=O, P for oxidation and phosphorylation depicted by the closed squares and closed circles respectively) for rat liver mitochondria with succinate as substrate as a function of time during the state 4 to state 3 transition [10,11]. The error bars lie within the symbol sizes selected.

**Figure 2:**
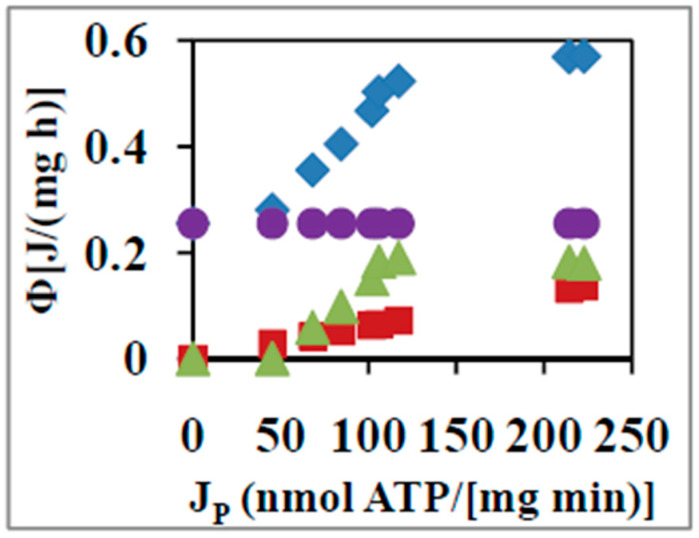
Parsing of the total rate of free energy dissipation into its various components for oxidative phosphorylation (OX PHOS) on succinate. $\Phi_{Total}$ (closed diamonds), $\Phi_{ATP\;synthesis}$ (closed squares), $\Phi_{leak}$ (closed circles), and $\Phi_{slip+other\;losses}$ (closed triangles) as a function of the rate of ATP synthesis, $J_{P}$ [10,11]. Error bars are contained within the selected symbol sizes.

Figure 1 shows the $J_{i}A_{i}$ components, where i=O, P for oxidation and phosphorylation respectively for the above system as a function of time during the state 4 to state 3 transition. Figure 2 plots the partitioning of $\Phi_{Total}$ among its elementary reactions or transport steps with respect to the variation of $J_{P}$ from the static head state 4 (where $J_{P}=0)$ to state 3 at which physiological ATP synthesis occurs and $J_{P}$ reaches its maximum or saturation value. How these calculations are made is described below.

From measurements reported in previous studies [10], the thermodynamic fluxes, i.e., the reaction rates of phosphorylation and oxidation, $J_{P}$ and $J_{O}$, were available at state 4 (static head) and at every temporal point as well as at each thermodynamic force of phosphorylation, $A_{P}$, during the transition from an initial state 4 to a final state 3 at which a maximal operating P/O ratio of oxidative phosphorylation was reached. The mechanistic P/O ratio, i.e., the maximum, zero loss stoichiometry for succinate oxidation, is known from structural data [42,43] and from nonequilibrium thermodynamic analysis [9]. For a detailed account of the basic distinction between these two stoichiometries, see reference [44]. The rate of oxygen consumption for ATP synthesis by the F_1_F_O_-ATP synthase (nmol mg^−1^ min^−1^) can be determined by division of the observed phosphorylation rate by the mechanistic P/O ratio. The resting static head or basal respiration rate, available from the data, is a measure of losses due to leak. A molecular model of the process has been formulated [9,10,44].

According to Nath’s two-ion theory of energy coupling [20,45], mitochondrial leak corresponds to the cycling across the coupling membrane of the uncharged form [H_2_A] of succinic acid [9,10,44]. This rate of oxygen consumption is required to maintain homeostasis of the neutral [H_2_A] species of the dicarboxylic acid along with homeostasis of [HA^−^], the motive form to which the ATP synthase is permeable, and the [A^2−^] species that contributes to redox slip during steady state operation. Hence from these considerations, the same basal rate of leak continues during the state 4 to state 3 transition into state 3. This answers a longstanding question in the field that has caused several headaches to researchers (please see the last two paragraphs of this Section). By subtraction of the rate of oxygen consumption for ATP synthesis and the rate of oxygen consumption required to replenish the leak from the total rate of oxygen consumption at each point, the rate of oxygen consumed for the slip reactions and other transport functions can be readily calculated. The losses due to leaks and slips are responsible for uncoupling in OX PHOS. These losses cannot be completely eliminated for a dicarboxylic acid system (Figure 1 in reference [9]). They can however be minimized, and a minimization of these losses due to leak and slip, or equivalently, maximization of the F_O_-permeant motive [HA^−^] form of the dicarboxylic acid [10] yields the highest possible degree of coupling, q, at which the system can operate (see Section 3, Section 4, and Section 7). The situation has been greatly clarified by the identification recently of the key variables that govern the efficiency of the process (see Sections 2.3 and 3.4 in reference [44]).

The free energy dissipation for the leak at a constant affinity of oxidation of succinate, $A_{O}$, of 160 kJ mol^−1^ is shown in Figure 2 by the closed circles. The rate of free energy utilized for ATP synthesis is readily obtained from the corresponding rate of oxygen consumption times the free energy of 58.7 kJ mol^−1^ required to synthesize an ATP molecule [9,10,45]. This is shown in Figure 2 by the closed squares. $\Phi_{Total}$ is readily evaluated from $\underset{i}{\sum }J_{i}A_{i}$ in which the relevant affinity of phosphorylation is now the complete thermodynamic function, inclusive of the concentration term involving ATP, ADP, and Pi [10]. This is depicted as a function of $J_{P}$ by the closed diamonds in Figure 2. By subtraction from $\Phi_{Total}$ of the Φ due to ATP synthesis and leak, the free energy dissipation due to slip and other losses is found at each value of $J_{P}$ (closed triangles in Figure 2).

The above analysis explains the modalities of calculation of $\Phi_{Total}$ as a function of output thermodynamic flux, $J_{P}$, and the partitioning of $\Phi_{Total}$ into the rate of free energy dissipation of its component reactions of ATP synthesis, leak, slip, and other losses (Figure 2). An analysis of $\Phi_{Total}$ as a function of time, quantification of the evolution of total and average unit action with time for the coupled biomolecular system, and an insightful interpretation in terms of the principle of least action has been made recently [11].

It had long been recognized since the pioneering bioenergetics studies of Kalckar, Ochoa, and Chance that mitochondrial preparations consume oxygen even when they are not synthesizing ATP [46,47,48,49]. It has been stated that “whether this basal oxygen consumption should be subtracted from the total oxygen consumption during ATP synthesis is an old question” [50]. However no answer to the dilemma was provided, and no rationale for deciding between the alternatives for a final resolution of this fundamental issue has been given to date. One group of workers uses the *total* oxygen consumed during the synthesis of ATP from added ADP to obtain the P/O ratio, presuming that the basal respiration rate continues during ADP phosphorylation in state 3 [51]. Another group of workers believes that “the best approximation for calculation of the P/O ratio is obtained by assuming that the state 4 rate of respiration ceases during ADP stimulated respiration” [52]. Thus the *extra* oxygen consumed in excess of state 4 respiration is also used in the calculation of the P/O ratio, and it is believed by some workers that unless this subtraction is made, the experimentally determined P/O ratios are lower than the values quoted in the biochemistry textbooks.

Nath’s two-ion theory of energy coupling [20,32,34,36,45] within Nath’s torsional mechanism of energy transduction and ATP synthesis [18,19,20,26,27,28,30,35,38] helps us to provide a definitive answer to an “old question” and to assess the final P/O stoichiometries of ATP synthesis [9,44]. By providing a detailed physicochemical framework [9,10,11,18,19,20,23,24,25,26,27,28,29,44,45] the theory helps us answer the “how and why” of the coupled nonequilibrium processes of ATP synthesis. Previously, Lee and co-workers had editorialized that “what is needed for assessing the final, mechanistic values is an elucidation of the molecular mechanisms of ion translocation and ATP synthesis through the various catalysts involved. Furthermore, the nature of the resting state 4 respiration of mitochondria and of energy-requiring reactions that compete with ATP synthesis under various experimental, physiological, and pathological conditions requires further research” [53]. It is hoped that exactly two decades after it was first formulated, and following its subsequent logical development and continued embellishment over the years, the availability of the now aged and detailed torsional mechanism of ATP synthesis will help catalyze the progress of research in the interdisciplinary field.

## 3. Calculation of the Thermodynamic Efficiency, η, from the Rate of Entropy Production

The thermodynamic efficiency, η, for the coupled nonequilibrium process of ATP synthesis can be calculated by the equation derived from first principles of linear nonequilibrium thermodynamics [4,6,13,54,55]

(2) $$\eta =1-\frac{\Phi }{J_{O}A_{O}}$$

The thermodynamic efficiency given by Equation (2) explicitly reveals its dependence on the *rates* at which the coupled nonequilibrium processes occur and thus differs from conventional definitions of efficiency in equilibrium thermodynamics. However, it ought to be stressed that in all nonequilibrium biothermodynamic studies to date, η has been defined as being equal to $-J_{P}A_{P}/\left(J_{O}A_{O}\right)$, where $A_{P}$ is taken as the *complete* thermodynamic function, i.e., including the $RTln\left(\frac{\left[ATP\right]}{\left[ADP\right]\left[Pi\right]}\right)$ term. We return to this important point in Section 7.2.

Equation (2) also shows that it is not necessary for Φ to decrease in order to produce an increase in η as assumed by some workers [56], given that $J_{O}$ is not a constant. The extent to which $J_{O}$ increases with increases in Φ determines whether η increases or decreases as Φ is increased. In fact, analysis of the data shows that Φ increases only ~50% on average compared to the increase in $J_{O}A_{O}$ during the state 4 to state 3 transition [10]. Thus, although Φ increases as we approach state 3 (Figure 2), $\Phi /\left(J_{O}A_{O}\right)$ decreases (Figure 1 and Figure 2), and therefore η increases in this range, and an overall thermodynamic efficiency for the coupled OX PHOS process on succinate of ~57% is estimated from the experimental data in state 3 at which physiological operation takes place [10,11].

The above considerations along with the experimental results [10,11] show that at the maximal (saturating) concentration gradients of ADP (outside to inside) and ATP (inside to outside) in state 3, given the constraint that availability of ADP is not limiting, and that the degree of coupling, q, of the system is kept constant (Section 4 and Equations (3) and (4)), then both $J_{P}$ and the actual operative P/O ratio (i.e., $J_{P}/J_{O}$) exhibit a maximum. Furthermore, Φ *as well as* η *exhibit a maximum* at the operating state 3 affinity force ratio subject to the above constraints. Needless to say, if q is increased (e.g., by reducing leaks or slips) or the concentration gradients of ADP and ATP are increased, then the system will shift to a new higher value of the maximum in these output functions. The optimal performance of linear energy converters is discussed further in Section 7.

## 4. Linear Nonequilibrium Thermodynamic Analysis of ATP Synthesis

Jou and Ferrer [2] have summarized the experimental values of the bulk Onsager phenomenological coefficients and leak conductance for redox-coupled ATP synthesis with succinate as substrate. Performing a linear nonequilibrium thermodynamic analysis for a steady state 2 × 2 i.e., a two flux, two force coupled system (Section 5 and Section 6) using these values [10] after incorporating 10% redox slip leads to a degree of coupling, q, of 0.97 and a phenomenological stoichiometry, Z, of 2.05. It should be stressed that the phenomenological stoichiometry, Z, obtained from a nonequilibrium thermodynamic analysis is not *a priori* identical to the mechanistic stoichiometry of the coupled OX PHOS process. However, if it is ensured that the bulk phenomenological coefficients have been expressed in terms of *elemental* parameters, and all key variables and interactions have been included in the analysis, then it is a reasonable expectation that Z ought to be a very good approximation of the mechanistic stoichiometry.

The well-known linear nonequilibrium thermodynamic equations for the operating P/O ratio and the thermodynamic efficiency, η, of the coupled process are [2,4,6,13,54]

(3) $$\frac{J_{P}}{J_{O}}=\frac{Z\left(q+Zx\right)}{1+qZx}$$

(4) $$\eta =-\frac{Zx\left(q+Zx\right)}{1+qZx}$$

where $q=L_{OP}/\sqrt{L_{OO}L_{PP}}$ and $Z=\sqrt{L_{PP}/L_{OO}}$ and $x=A_{P}/A_{O}$.

Substituting the values of the kinetic parameters q and Z into Equations (3) and (4) for the operating state 3 affinity force ratio, $x=A_{P}/A_{O}$ of −0.36 with succinate as substrate [10,11,57], the linear nonequilibrium thermodynamic analysis yields an actual P/O ratio of 1.55, in agreement with experimental determinations [10,44] and a thermodynamic efficiency, η, of 57%, in consonance with the value obtained in Section 3. Under the prevailing constraints the rate of free energy dissipation, Φ *also exhibits a maximum* [10,11]. The value of the state 4 affinity force ratio at which the phosphorylation flux vanishes is given by q/Z, which works out to be −0.47. This maximal affinity force ratio at static head deviates from the experimentally determined value of ~−0.40 for the process with succinate as substrate [10]. The reasons for this deviation will be clear only when the underlying molecular mechanisms are understood and included in the nonequilibrium thermodynamic analysis that requires a 3 × 3 matrix with the phenomenological coefficients obeying Onsager symmetricity (see Section 8.1 and Section 8.2).

## 5. Derivation of the Degree of Coupling, q, between Oxidation and ATP Synthesis from Experimental Data Using Nonequilibrium Thermodynamics

The degree of coupling, q, can also be derived by applying the experimental information to a general relationship arising from linear nonequilibrium thermodynamics for a 2 × 2 system. Specifically, we need to know the respiratory control ratio (RCR) from the experiment, which is the ratio of the rates of respiration in state 3 and state 4, and the phosphorylation affinities in states 3 and 4. For a steady state of zero net ionic flux, the phenomenological equations can be written as

(5) $$J_{O}=L_{OO}A_{O}+L_{OP}A_{P}$$

(6) $$J_{P}=L_{OP}A_{O}+L_{PP}A_{P}$$

where the L symbols represent the symmetric matrix of the Onsager phenomenological conductances of oxidation, ATP synthesis, and of the overall coupling between the two nonequilibrium processes. Applying the condition of disappearance of the phosphorylation flow in state 4 in Equation (6) we obtain

(7) $$L_{OP}=-\frac{L_{PP}A_{P,\;state\;4}}{A_{O}}$$

Substituting Equation (7) into Equation (5) we have

(8) $$J_{O,\;state\;4}=L_{OO}A_{O}-\frac{L_{PP}A_{P,state\;4}^{2}}{A_{O}}$$

(9) $$J_{O,state\;3}=\frac{L_{OO}A_{O}^{2}-L_{PP}A_{P,state\;3}A_{P,state\;4}}{A_{O}}$$

Equation (8) can be written as

(10) $$J_{O,state\;4}=L_{OO}A_{O}\left(1-\frac{L_{PP}A_{P,state\;4}^{2}}{L_{OO}A_{O}^{2}}\right)$$

By definition,

(11) $$q^{2}=\frac{L_{OP}^{2}}{L_{OO}L_{PP}}$$

Substituting Equation (7) into Equation (11) we have

(12) $$q^{2}=\frac{L_{PP}A_{P,state\;4}^{2}}{L_{OO}A_{O}^{2}}$$

Using Equation (12) in Equation (10) we obtain the rate of oxygen consumption in state 4 as

(13) $$J_{O,state\;4}=L_{OO}A_{O}\left(1-q^{2}\right)$$

From Equations (9) and (13) we obtain

(14) $$RCR=\frac{J_{O,state\;3}}{J_{O,state\;4}}=\left(1-\frac{L_{PP}A_{P,state\;3}A_{P,state\;4}}{L_{OO}A_{O}^{2}}\right)\times \frac{1}{1-q^{2}}$$

or using Equation (12) in Equation (14)

(15) $$RCR\left(1-q^{2}\right)-1=-\frac{L_{PP}A_{P,state\;3}A_{P,state\;4}}{L_{OO}A_{O}^{2}}=-q^{2}\frac{A_{P,state\;3}}{A_{P,state\;4}}$$

which upon rearrangement leads to the final equation

(16) $$q^{2}=\frac{RCR-1}{RCR-\frac{A_{P,state\;3}}{A_{P,state\;4}}}$$

with $RCR=\frac{J_{O,state\;3}}{J_{O,state\;4}}$.

From the experimental data (Figure 2 and reference [10]), RCR measures five for the system. $\frac{A_{P,state\;3}}{A_{O}}$ measures −0.36 from the data. Using $\frac{A_{P,state\;4}}{A_{O}}=-0.47$ from the linear nonequilibrium thermodynamic analysis (Section 4) in Equation (16) we arrive at a value of the degree of coupling, q, for the OX PHOS process on succinate of 0.97, which matches well with the value obtained in Section 4.

## 6. Derivation of a Relationship for ADP/([Extra O]) in Terms of Kinetic Parameters and Comparison with Experimental Results

The thermodynamic analysis in Section 4 and Section 5 has employed the ADP/TOTAL O. We now derive a relationship for the ADP consumed at any temporal point during the state 4 to state 3 transition divided by the extra oxygen consumed, i.e., the oxygen consumed in excess over state 4 respiration. The final Equation (20) has previously been derived by Lemasters [58] who convincingly demonstrated that nonequilibrium thermodynamics can be utilized to provide important new information on the OX PHOS process. However, we present an alternative derivation here.

(17) $$\left[EXTRA\;O\right]=\;J_{O}-J_{O,state\;4}$$

Substituting from Equation (5) and Equation (13) in Equation (17) we get

$$\left[EXTRA\;O\right]=\left(L_{OP}A_{P}+L_{OO}A_{O}\right)-L_{OO}A_{O}\left(1-q^{2}\right)$$

$$=L_{OO}A_{O}\left(1+qZ\frac{A_{P}}{A_{O}}\right)-L_{OO}A_{O}\left(1-q^{2}\right)$$

i.e.,

(18) $$\left[EXTRA\;O\right]=L_{OO}A_{O}q\left(q+Z\frac{A_{P}}{A_{O}}\right)$$

Now for the Chance–Williams type experiment [48,49], from Equation (6), the rate of ADP consumption

(19) $$\dot{\left[ADP\right]=}J_{P}=L_{OP}A_{O}+L_{PP}A_{P}=ZL_{OO}A_{O}\left(q+Z\frac{A_{P}}{A_{O}}\right)$$

Combining Equations (18) and (19), we arrive at the final result

(20) $$\frac{J_{P}}{\left[EXTRA\;O\right]}=\frac{Z}{q}$$

Applying Equation (20) to state 3 conditions of ADP phosphorylation yields $Z/q=\frac{2.05}{0.97}=2.1$ which is in reasonably good agreement with the final state 3 experimental value of $J_{P}/\left[EXTRA\;O\right]=J_{P}/\left(J_{O,state\;3}-J_{O,state\;4}\right)=2.03$ (Figure 1 and reference [10]).

## 7. Optimality of Biological Free Energy Converters in OX PHOS

From the scientific literature on the subject of optimization of linear energy converters [4,13,54,55,59], it is not obvious if the optimal state of a biological free energy converter is one of maximum efficiency. It may be necessary in some systems to produce the maximum output flux. In emergency situations, for example in predator–prey cases where the organism is trying to flee, generation of a maximum output power may prove to be the difference between survival and death. Other converters may be optimal if the product of efficiency and output flow, termed economic output flow is maximal, or alternatively if the product of efficiency and output power, (termed maximum economic output power in the literature [13]) is maximal. These optimality functions are defined again in Table 1 for the sake of clarity. We can carry out a single variable optimization by expressing these functions in terms of the degree of coupling, q, and the normalized force ratio, $Zx=ZA_{P}/A_{O}$, fixing q at its maximal value of 1, and determining the value of Zx at which the output function is maximal. Substitution of this value of Zx and taking q=1 in Equation (4) yields the value of the thermodynamic efficiency in percentage (Table 1) so that the desired output function is maximal.

However, such a calculation is not of practical value because all known biological energy converters are uncoupled to an extent, i.e., the degree of coupling is less than 1. Moreover, no single universal value of q has been found. Further, the calculation (Table 1) provides no explanation as to why the coupling is less than perfect.

In his seminal work, Stucki [13] proposed a two-variable optimization, and considered both the *normalized* force ratio as well as the degree of coupling as variables. The implication was that during the course of evolution, or because of the process of metabolic regulation, both these variables would have been adjusted to reach their optimal value. The theoretical thermodynamic efficiency was calculated (Table 1) when the output function was maximized by varying with respect to the normalized force ratio, while the efficiency was optimized by adjusting the degree of coupling to the normalized force ratio [13]. Note that in the calculations the output functions of maximum output flow, maximum output power, maximum economic output flow, and maximum economic output power (Table 1) were normalized by the q−dependent factors $ZL_{OO}A_{O},\;L_{OO}A_{O}^{2},\;ZL_{OO}A_{O},$ and $L_{OO}A_{O}^{2}$ respectively.

The idea that both output phosphorylation affinity, $A_{P}$, (or the affinity force ratio, $x=A_{P}/A_{O}$) and the degree of coupling, q, may be varied by the coupled biological system in order to achieve optimal performance is very important and interesting. Firstly, Stucki’s way of variation is not the only possible one. In a nutshell, Stucki’s solution [13] to the problem was to adjust the affinity force ratio for each and every possible degree of coupling in order to yield the maximum efficiency after which the degree of coupling is adjusted to produce a maximum value of a second output function (Table 1 and Table 2). This operational procedure leads to the degree of coupling tabulated in the second column of Table 2. However, if the order in which the optimization processes are carried out is reversed, or if the variables in the optimization process are interchanged, the results are very different and lead to a q of 1 and a flux ratio that is always equal to the phenomenological stoichiometry, Z, and is furthermore invariant with respect to the force ratio in the entire range.

There are several other lacunae in Stucki’s analysis, pointed out recently [44]. A necessary and sufficient condition for the validity of Stucki’s results is the satisfaction of his “conductance matching” condition (Equation (36) in reference [13]) which has not been proven. Further the work postulates an optimum of *minimum* Φ, although it does not meet the conditions of Prigogine’s theorem of vanishing flow at static head for which the minimum condition was derived [8]. Moreover, analyses by other researchers have led to the opposite result of a *maximum* Φ, under the given constraints [10,11,14]. Starting from first principles, Martyushev [60,61] has given a general proof for the latter, which strengthens the arguments made here. Moreover, Z varies with q, a fact that was ignored in Stucki’s analysis. In fact, in actual experiments we measure a non-normalized affinity force ratio, x, and only subsequently do we calculate a value of Z. Hence there appears to be no basis for normalization of the output functions by various q-dependent factors (Table 1). We have carried out a refined analysis based on the *unnormalized* force ratio (Table 2). We find that the optimal q depends on whether the output function is normalized or not. Moreover, the mechanism of uncoupling also affects the value of the degree of coupling, q, in the optimal state (Table 2). In particular, if the optimized function is output flow, the difference between the two analyses is very drastic. Finally, the state 4 static head force ratio shows very little variation with q for the non-normalized case compared to the normalized case, because in the latter case, the normalized force ratio, Zx, is exactly equal to q.

Based on the above analysis, we offer two possible approaches for arriving at the optimality criteria for biological free energy converters, (i) the standard approach based on biothermodynamics, and (ii) an alternative that includes (i) but possesses additional features of interest. We have named it a *biothermokinetics* approach. We summarize these approaches in Section 7.1 and Section 7.2.

### 7.1. Biothermodynamics Approach to Optimality Criteria for Free Energy Converters in OX PHOS

The biothermodynamics approach has been described above. Application of this approach to the coupled nonequilibrium processes of ATP synthesis led to a degree of coupling, q, of 0.97 (Section 4 and Section 5). From the results summarized in Table 2 for the refined biothermodynamics approach based on *non-normalized* output functions, this value of q of 0.97 shows that the linear biological free energy converters of OX PHOS are tuned for the maximization of economic output power, $J_{P}A_{P}\eta$.

**Figure 3:**
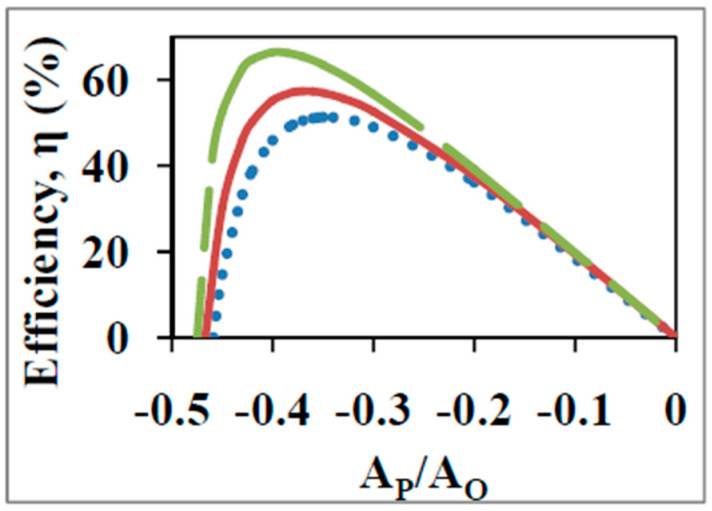
Efficiency of energy conversion, η vs. the non-normalized force ratio $x=A_{P}/A_{O}$ with the degree of coupling, q, as a parameter for ATP synthesis on succinate as calculated from the standard biothermodynamic approach. q was varied by varying the mitochondrial leak coefficient and equals 0.98 (dashed line), 0.97 (bold line), and 0.95 (dotted line).

A major difficulty with the purely biothermodynamics approach lies in the fact that for the four output functions tabulated in Table 2, i.e., maximum output flow, maximum output power, maximum economic output flow, and maximum economic output power, the optimal degree of coupling increases from 0.786 to 0.909 to 0.953 and 0.973 respectively. Thus, the finding of one of these values of q for the biological energy converter implies an optimization of one of these output functions at the expense of the others. Moreover, the optimal value of Zx at which each of these four functions is optimal also varies from −0.486 to −0.644 to −0.732 to −0.786 respectively. In other words, the optimal normalized force ratio, |Zx|, shifts to higher absolute values as q increases, corresponding to an increased ATP/ADP ratio, i.e., to progressively lower ADP concentrations. Similar results are obtained for the various cases without normalization, and Figure 3 demonstrates this for the η vs.x curves with q as a parameter for ADP phosphorylation on succinate (Section 2 and Figure 1 and Figure 2). In Figure 3 the degree of coupling was varied by varying the mitochondrial leak. It was found that there is a shift to higher |x| with increase in q. This is a leftward shift in Figure 3 to lower ADP concentrations as the degree of coupling and thermodynamic efficiency increases. Moreover, as shown in previous sections, shifts to higher ADP concentrations outside and lower ATP concentrations outside lead to increased thermodynamic efficiencies, given especially the rate limiting nature of the availability and concentration gradient (outside to inside) of ADP. Hence shifts to the right in |x| (Figure 3) should have led to increased efficiencies of energy transfer, η. In other words, it should have been possible for the system to shift to the right and obtain a further increase in η at larger q values. However, the opposite is observed (Figure 3). What could be the underlying reasons behind this discrepancy? This is treated in Section 7.2.

### 7.2. Biothermokinetics Approach to Optimality Criteria for Free Energy Converters in OX PHOS

**Figure 4:**
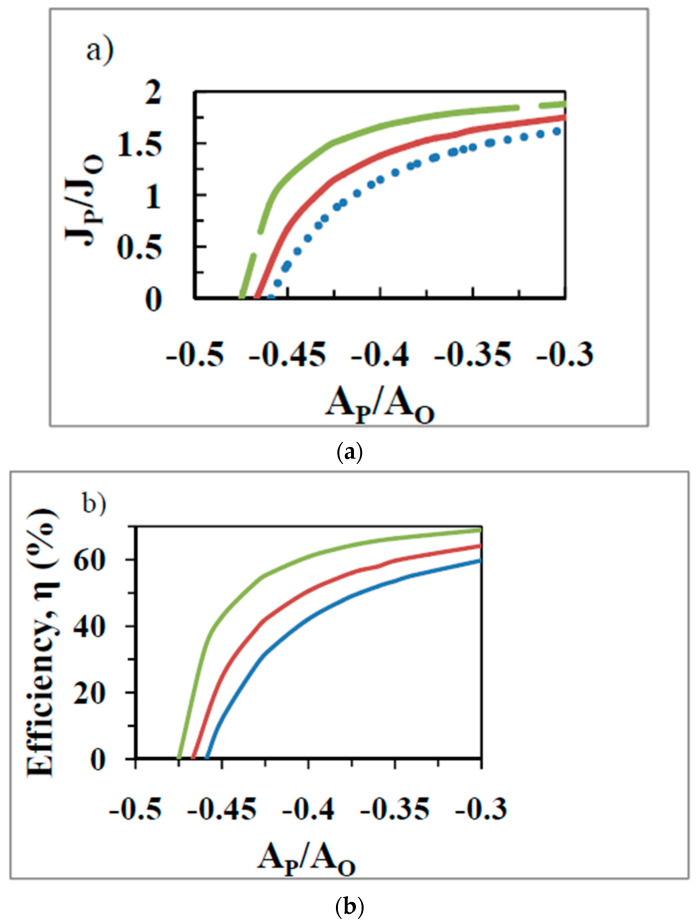
The flux ratio $J/J_{O}$ (**a**) and the efficiency, η, of ATP synthesis (**b**) as a function of the force ratio $x=A_{P}/A_{O}$, with the degree of coupling, q, as a parameter calculated by the biothermokinetic approach proposed in this work. q was varied by varying the mitochondrial leak coefficient and equals 0.98 (top curve), 0.97 (middle curve), and 0.95 (bottom curve).

As discussed in Section 7.1, in the purely biothermodynamic approach, the optima in the various output functions of OX PHOS (Table 1 and Table 2) are realized at *different* values of the force ratio. The question then arises that at the value of the operating force ratio (x=−0.36 in Figure 1, Figure 2 and Figure 3 with succinate and x=−0.266 for NADH-based substrates [9,10,11,57]) for what performance function is the biological system optimized given the constraint (at high Pi concentrations) of the limiting ADP concentration gradient (inwards) and the ATP concentration gradient (outwards) in the mitochondrion, or since only the external concentrations are normally measured, of the operating ATP/ADP ratio, which is an approximation of the former true driving force (concentration gradients) for transport of ADP and ATP metabolites? In the biothermodynamic framework, it is not possible to *simultaneously* realize maximal output flux and maximal efficiency (compare Figure 3 with Figure 4a), or maximal output power and maximal efficiency, or maximum efficiency and maximum Φ etc., at the operating force ratio, as shown in Section 7.1. Hence in this framework, one is forced to choose a particular output function as the aim of biological energy converters that carry out these fundamental processes. For OX PHOS, this was shown above to be maximization of economic output power, $J_{P}A_{P}\eta$, by the double optimization process with q=0.97.

In order to energetically support the network of living systems and meet the energy demands of the cell, it is a crucial function of mitochondria to guarantee a maximal or at least a sufficiently high ATP flux. However, unlike the efficiency or the output power (as modeled by the biothermodynamic approach), the output flow, $J_{P}$, and the output flow ratio $J_{P}/J_{O}$ do not exhibit a maximum. The values of these latter functions keep increasing with decreases in |x| (Figure 4a) at every positive value of the degree of coupling. Hence for these functions presumably the optimal state is one at which the value of |x| is as small as possible consistent with other limitations imposed on the system.

To a very large extent, the source of the difficulties mentioned above and in Section 7.1 lie in the very definition of the thermodynamic efficiency of ATP synthesis, η (see text accompanying Equation (2)). At first sight, it appears correct to define the efficiency as the ratio of the output free energy rate divided by the input free energy rate, with $A_{P}$ considered as the complete thermodynamic function, i.e., with the $RTln\left(\frac{\left[ATP\right]}{\left[ADP\right]\left[Pi\right]}\right)$ term, the so-called phosphate potential added to a standard state term. However, this assumes inadvertently that the mechanism of the chemical reaction on the enzyme and the mechanism of substrate and product transport through the transporters are similar and are dependent on the same variables, and specifically that both are governed by the complete affinity term. In other words, it has been tacitly assumed that the rate of the reaction and its energetics are both governed by the complete thermodynamic function, $A_{P}$. However, while the ADP, Pi, and ATP kinetics and overall transport rates are indeed proportional to the concentration gradients of these metabolites, or in the biothermodynamic framework, governed by the change in chemical potential of these metabolites as modeled by the concentration dependent term $RTln\left(\frac{\left[ATP\right]}{\left[ADP\right]\left[Pi\right]}\right)$ in the definition of $A_{P}$, there is no reason for the energetics and the output free energy in a *molecular* energy transduction process on the F_1_F_O_-ATP synthase to be so governed.

The central arguments made above imply that the rate of substrate, product, or ion transport is indeed governed by the corresponding metabolite or ion concentration gradient. However, in the act by act molecular processes of energy transduction catalyzed by the ATP synthase enzyme, the energy donated per discrete, quantized act of ion translocation is the same irrespective of the concentration gradient or electrochemical potential difference of that ion. In other words, as far as the single molecule of ATP synthase is concerned, in the molecular process of energy transduction, it uses a constant free energy, $\Delta G_{P}=-A_{P}$ of 58.7 kJ/mol to synthesize an ATP molecule [9,10,11,28,44,45]. Furthermore, the energy extracted per H^+^ ion translocation through the access channels in the membrane-bound F_O_ portion of the F_1_F_O_-ATP synthase enzyme is the same, irrespective of the gradient of the various species/ions, and thus the relevant potentials are not the complete thermodynamic affinities.

Thus defined, the $A_{O}/A_{P}$ value measures the maximum number, n, of ATP molecules that can be synthesized per oxygen atom consumed and therefore the ratio of the operating P/O to n is already a measure of the thermodynamic efficiency, η, of ATP synthesis [44], as it is the ratio of the actual P/O to the mechanistic P/O. The actual P/O is lowered from its maximal mechanistic value due to the inefficiencies arising from leaks, slips, side reactions, and other losses in the system. Thus, a properly formulated biothermokinetic scheme automatically takes care of the thermodynamics also.

Several other kinetic arguments and molecular reasons can be given in support of the above biothermokinetic viewpoint. The influence of the ADP, Pi, and ATP concentrations has already been accounted for in the phosphorylation rate, $J_{P}$, so there is no reason to account for it a second time in $A_{P}$. For very high ADP transport rates as would occur if extramitochondrial ADP concentration is increased beyond a certain range, the ATP pumps can undergo slippage, lower the degree of coupling, and fail to operate efficiently below a critical value of |x|. This conclusion is strengthened by our experimental finding that it was impossible to lower the phosphate potential below a certain value (|x|<0.25 for NADH-based substrates). We attribute this to the buffering activity of myokinase, ensuring that the force ratio remains close to its value in the optimal state (|x|=0.266) for such substrates.

From the classical biothermodynamics viewpoint (Section 7.1), lowering |x| below the physiological state 3 value of |x|=0.266 for NADH-based substrates (|x|=0.36 for succinate) leads to a provision of energy <58.7 kJ/mol required to make an ATP molecule. Hence there is insufficient energy to synthesize ATP, and therefore such a region of operation is not observed in practice. A higher |x| leads to the provision of >58.7 kJ/mol to synthesize a single molecule of ATP, and implies that the excess energy above the required quantum of 58.7 kJ/mol is not usefully employed. In other words, operation in this region is sub-optimal with respect to thermodynamic efficiency of the process, as found (Figure 4b).

However, the strongest argument that can be brought to bear in support of the biothermokinetic viewpoint is molecular in nature. The kinetically rate limiting nature of the ADP and ATP transport steps in the overall ATP synthesis process occur through the adenine nucleotide translocase (ANT), while the chemical ATP synthesis reaction takes place on a different complex, i.e., the F_1_F_O_-ATP synthase localized across the cristae membrane, which is spatially separated from the ANT located at the inner mitochondrial membrane contact sites with the outer membrane. Hence there is no reason for them to be governed by identical driving forces. Simplistically we can say that the variable $\left(\frac{\left[ATP\right]}{\left[ADP\right]\left[Pi\right]}\right)$ is associated with the functioning of ANT during the rate limiting transport of ADP in and ATP out of the mitochondrion in the overall process of ATP synthesis, while the constant $A_{P}$ is associated with the F_1_F_O_ during the actual chemical synthesis step. This spatial and mechanistic separation permitted the observation simultaneously of a maximum output flux, maximum output power, maximum efficiency, and maximum free energy dissipation at a particular (optimal) value of the force ratio. However, this separation was not reflected in the standard biothermodynamic analysis which used the same thermodynamic function, and assumed that the equations containing the complete thermodynamic term were applicable to model both the transport as well as the energetics of the chemical reaction. Such a seemingly unimportant omission however had grave and unforeseen consequences, as discussed in detail in this section. Knowledge of the underlying molecular details formulated by Nath’s two-ion theory of energy coupling within the torsional mechanism of ATP synthesis greatly helped in reaching a deeper fundamental understanding of energy coupling in these processes.

The biothermokinetic analysis is represented in Figure 4a,b for OX PHOS with succinate as the respiratory substrate. The degree of coupling is varied by altering mitochondrial leak by using different values for the passive leak conductance of the membrane. The operating state 3 force ratio in these diagrams for succinate is −0.36. Because of the use of a constant output affinity of phosphorylation, the curve for efficiency (Figure 4b, unlike that in Figure 3 for the standard biothermodynamic analysis) is now similar to the curve for the output flux ratio (Figure 4a). For a constant x, increase in q leads to an increased P/O ratio and increased η (vertical movement in Figure 4 at constant $A_{P}/A_{O}$). In order to obtain the same P/O ratio and η at a lower value of q, it is required to increase the external ADP and/or Pi concentrations and/or lower the external ATP concentration (horizontal movement rightward in Figure 4 at constant $J_{P}/J_{O}$). For a particular value of q, the hyperbolic dependence predicted between P/O vs. force ratio or efficiency vs. force ratio is found (Figure 4), as observed during the state 4 to state 3 transition, and also predicted from the nonequilibrium thermodynamics. This is due to the increase in phosphorylation and respiration rates as |x| decreases, given the existence of a constant state 4 respiration rate that persists into state 3, leading to a hyperbolic relationship between $J_{P}/J_{O}$ and x (Section 2 and Figure 4a).

To summarize, a high (maximal) concentration gradient of ADP into the organelle that determines the physiological operating force ratio in state 3 is responsible for a high (maximal) phosphorylation flux that almost saturates at high extramitochondrial ADP concentrations for values of the degree of coupling approaching 1 (Figure 4). This produces *simultaneously, at the same force ratio,* the maximum efficiency, the maximum output power, and the maximum entropy production rate, given an upper limit to the ADP concentration in the system. It can be readily visualized that this upper limit of ADP concentration that fuels the demand for ATP production by mitochondria is governed by the net ATP utilizing processes in the cell during steady state functioning [44]. The minimization of leaks, slips, and other losses leads to as high a degree of coupling as possible by optimization of intracristal and matrix space variables [9,10], and this enhances all the four output functions *independently* of the former effect. This double optimization explains the performance of free energy converters in the biothermokinetic process of OX PHOS.

## 8. Molecular Mechanisms Beyond the Chemiosmotic Theory

### 8.1. Experiments on ADP Phosphorylation During the Transition from State 4 to State 3 or in State 3

The phenomenon of respiratory control as demonstrated by a high RCR ratio is one of the key fundamental experimental observations that relates to the molecular mechanism of OX PHOS. The control exercised by the phosphorylation reaction on the rate of respiration strongly indicates the presence of *direct* coupling between the two reactions. The process of uncoupling of ion transport from ATP synthesis when the control of the ATP reaction on respiration is weakened or completely released also shows the importance of coupling in control of respiration rates and the role of energized ionic intermediates in the coupling process. Any model of OX PHOS has to be able to explain both these phenomena in a compatible way.

The classical chemiosmotic theory [15,16] predicts that for any value of the respiration rate $J_{O}$ there corresponds a unique value of $\Delta {ũ}_{H}$. Thus, an equal stimulation of respiration over state 4 respiration by either ADP or uncouplers is predicted to correspond to an equal reduction of $\Delta {ũ}_{H}$ by the theory. We searched for an uncoupling concentration of 2,4-dinitrophenol that yields the same rate of respiration as given by the phosphorylation system with ADP in a parallel experiment (Table 3). However, we found in our experiments that stimulation of respiration with 0.4 mM ADP (line 1 in Table 3) was associated with a decrease in $\Delta {ũ}_{H}$ from state 4 of only ~5 mV, while the same stimulation of respiratory rate could only be achieved by a reduction of $\Delta {ũ}_{H}$ by as much as 35–40 mV from state 4 when 2,4-dinitrophenol (3 μM) was used as an uncoupler (line 2 in Table 3). These experimental observations are incompatible with Mitchell’s chemiosmotic theory and cannot be explained by it.

Within the framework of linear nonequilibrium thermodynamics (Section 4 and Section 5), the processes of oxidation, ATP synthesis, and ion transport are driven respectively by the oxidation affinity $A_{O}$, the phosphorylation affinity $A_{P}$, and the electrochemical potential difference of ions (e.g., protons) involved in coupling $\Delta {ũ}_{H}$. Thus, the phenomenological Onsager equations representing oxidative phosphorylation can be written as a 3 × 3 symmetric matrix in terms of conductances as follows:

(21) $$J_{O}=L_{OO}A_{O}+L_{OH}\Delta {ũ}_{H}+L_{OP}A_{P}$$

(22) $$J_{H}=L_{OH}A_{O}+L_{HH}\Delta {ũ}_{H}+L_{PH}A_{P}$$

(23) $$J_{P}=L_{OP}A_{O}+L_{PH}\Delta {ũ}_{H}+L_{PP}A_{P}$$

**Figure 5:**
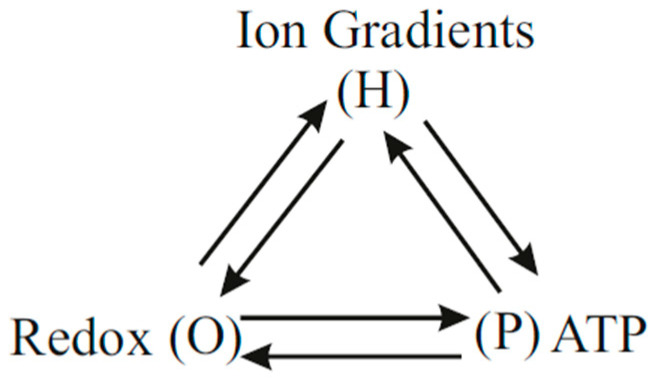
Coupling scheme in OX PHOS based on Nath’s two-ion theory of energy coupling in ATP synthesis.

Mitchell’s chemiosmotic theory postulates the absence of direct coupling between oxidation and ATP synthesis other than that mediated indirectly via the H^+^ pumps through the protonmotive force. Hence, for chemiosmotic coupling, $L_{OP}=0.$ Thus the chemiosmotic theory fails to explain the phenomenon of respiratory control in OX PHOS. According to Nath’s two-ion theory of energy coupling, the binding of the cycled succinate anions to the respiratory enzyme complexes activates respiration from the mitochondrial matrix side. An integrated model of OX PHOS based on the considerations in this work is given in the scheme of Figure 5. Each connection in Figure 5 indicates the existence of a coupling mechanism. The direct coupling is shown by the horizontal line in the scheme of Figure 5 and explains the control of the phosphorylation reaction on oxidation, and the phenomenon of respiratory control.

The above experimental observations (Table 3) are explained by the integrated model shown in Figure 5 because according to Nath’s two-ion theory of energy coupling and the torsional mechanism of ATP synthesis, in addition to the indirect coupling mediated by the so-called protonmotive force of chemiosmosis, the binding to the redox enzyme complexes in the respiratory chain of succinate ions translocated through the ATP synthase along with protons mediates a direct effect on respiration. This direct interaction does not require the cycling of protons across bulk aqueous phases. It can be modeled adequately by use of a finite positive value of $L_{OP}$ in Nath’s two-ion theory of energy coupling [20].

### 8.2. Experiments Probing State 4

As explained in Section 2, Section 4 and Section 5, respiring mitochondria phosphorylate added ADP until they reach a state 4 condition characterized by the absence of any further net ATP synthesis. According to Mitchell’s chemiosmotic theory, in this static head state 4, the reaction catalyzed by the ATP synthase has reached equilibrium. In this state, the phosphate potential, $\Delta G_{P}$, plus the H^+^/ATP stoichiometry of the synthase proton pumps, $n_{P}$, times the electrochemical potential difference of protons, $\Delta {ũ}_{H}$, equals zero. That is,

(24) $$A_{P}=n_{P}\Delta {ũ}_{H}$$

Thus the ratio of phosphate potential to $\Delta {ũ}_{H}$ is predicted by chemiosmosis to be constant and equal to the proton pump stoichiometry of the ATP synthase as follows

(25) $$\frac{A_{P}}{\Delta {ũ}_{H}}=n_{P}$$

**Figure 6:**
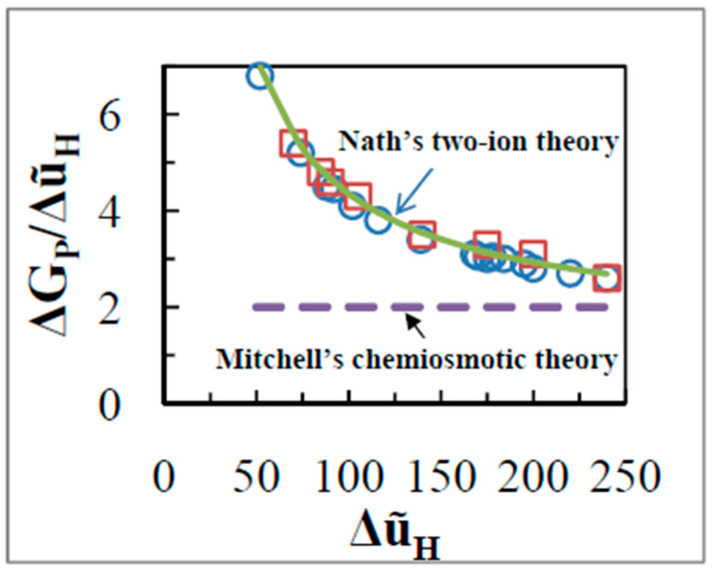
$\Delta G_{P}/\Delta {ũ}_{H}$ as a function of $\Delta {ũ}_{H}$ in state 4 (“static head”) with succinate as substrate. $\Delta {ũ}_{H}$ was varied with uncoupler FCCP (0 – 0.1 nmol/mg) (open circles) or by use of the redox inhibitor malonate (0 – 20 mM) (open squares). Error bars lie within the selected symbol sizes. The bold line shows the hyperbolic dependence of $\Delta G_{P}/\Delta {ũ}_{H}$ on $\Delta {ũ}_{H}$ in state 4 as predicted by Nath’s theory of energy coupling (Equation (28)).

According to Mitchell’s theory, the theoretical value of the above ratio should be constant at 2 [15]. However, in a titration with the uncoupler carbonyl cyanide p-trifluoromethoxyphenylhydrazone, FCCP (0 to 0.1 nmol mg^−1^), the ratio of $A_{P}/\Delta {ũ}_{H}$ was found to greatly exceed 2 and reach as high a value as 6 at low values of $\Delta {ũ}_{H}$ of ~50 mV (Figure 6). Most important, $A_{P}/\Delta {ũ}_{H}$ *varied hyperbolically* with $\Delta {ũ}_{H}$ (Figure 6), which is completely inconsistent with the prediction of the chemiosmotic theory (see Equation (25)). Similar results appear to have been first obtained by the group of Azzone (see Figure 6 of reference [62]), although they performed their experiments using different methods of measurement based on labeled tracers of ^86^Rb^+^, [^3^H]triphenylmethylphosphonium (TPMP^+^), ^45^Ca^2+^, or [^3^H]acetate. They clearly stated that “when $\Delta G_{P}$ and $\Delta {ũ}_{H}$ are depressed by uncouplers, the $\Delta G_{P}/\Delta {ũ}_{H}$ ratio increases hyperbolically tending to infinity while $\Delta {ũ}_{H}$ tends to zero” [62].

The same state 4 experiment was performed by titration with varying concentrations of malonate, an inhibitor of succinate dehydrogenase, and again a very similar dependence to that by uncoupler was found (Figure 6). The ratio of phosphate potential to $\Delta {ũ}_{H}$ was dependent on the $\Delta {ũ}_{H}$ in this case also, in contradiction with the prediction of the chemiosmotic theory. The data show that the variation of the state 4 ratio of $A_{P}/\Delta {ũ}_{H}$ on $\Delta {ũ}_{H}$ is independent of whether $\Delta {ũ}_{H}$ is varied by uncoupler or by variation of the activity of the respiratory chain (Figure 6).

The chemiosmotic theory, according to which energy transduction from oxidation to ATP synthesis occurs solely through a $\Delta {ũ}_{H}$ across bulk aqueous phases, is inconsistent with the results of both the uncoupler and malonate experiments shown in Figure 6, unless variable stoichiometry of the proton pumps is assumed. However, from structural and biochemical information, there is absolutely no basis to assume variable stoichiometry [42,43,63]. Moreover, the results of Figure 6 cannot be explained by a high energy chemical intermediate of unknown nature, $\Delta G_{X}$, that essentially replaces a physical high energy intermediate $\Delta {ũ}_{H}$ by a chemical one. Nor can the results be explained by a model in which the $A_{P}$ is converted to $\Delta {ũ}_{H}$ by a chemical reaction or by physical means, or by any scheme in which an energetic intermediate is in communication with the bulk-to-bulk $\Delta {ũ}_{H}$.

The integrated model given by the scheme of Figure 5 involving a *dual* mode of coupling postulated in Nath’s two-ion theory of energy coupling [20,44] is consistent with the results of both the uncoupler and malonate experiments shown in Figure 6. The hyperbolic relationship in Figure 6 can also be derived mathematically using Equation (23). Written for the static head state 4, this equation takes the form

(26) $$0=L_{OP}A_{O}+L_{PH}\Delta {ũ}_{H}-L_{PP}\Delta G_{P}$$

Or, using the relationship for $L_{PH}$ derived earlier [57] with $n_{O}$ and $n_{P}$ as the ion stoichiometries of redox and ATP-side pumps respectively,

(27) $$L_{PP}\Delta G_{P}=L_{OP}A_{O}+\left(n_{P}L_{PP}-n_{O}L_{OP}\right)\;\Delta {ũ}_{H}$$

i.e., we obtain the principal equation of this section modeling the energy relationships in state 4 according to Nath’s theory as

(28) $$\frac{\Delta G_{P}}{\Delta {ũ}_{H}}=\frac{L_{OP}A_{O}}{L_{PP}\Delta {ũ}_{H}}+\left(n_{P}-\frac{n_{O}L_{OP}}{L_{PP}}\right)$$

Equation (28) is of the form $y-k=k^{\prime }/x$ where $y=\frac{\Delta G_{P}}{\Delta {ũ}_{H}}$ and $x=\Delta {ũ}_{H}$ and k and $k^{\prime }$ are constants and exhibits a hyperbolic relationship of y on x as found in Figure 6, with $k^{\prime }$ as the gain or multiplier for the y-axis, and k as the asymptote at high $\Delta {ũ}_{H}$.

The value of the phenomenological coefficient directly coupling oxidation and phosphorylation, $L_{OP}$, obeys the inequality $L_{OP}^{2}\leq L_{OO}L_{PP}$ imposed by the second law of thermodynamics. Preliminary kinetic analysis of our data for OX PHOS on succinate yields a value for $L_{OP}$ of 1.0 ± 0.05 nmol mg^−1^min^−1^mV^−1^. This is considerably different from the implication of $L_{OP}=0$ in the chemiosmotic theory. In fact, Equation (28) in Nath’s theory provides a clear-cut illustration of the relevance of a positive, non-vanishing value of $L_{OP}$. In addition, the theory proposes that succinate, the *“second ion”* in Nath’s two-ion theory of energy coupling, is the elusive signaling molecule, X [20,44]. The binding of succinate to the enzyme complexes on the redox side determines the transport coefficient $L_{OP}$, thereby providing feedback for ATP energy supply and demand matching, and an explanation of the fundamental phenomenon of respiratory coupling in oxidative phosphorylation. Hence Nath’s two-ion theory of energy coupling in ATP synthesis, being microscopic in nature, also provides a molecular interpretation of the transport coefficient, $L_{OP}$.

Taken together, we conclude that the integrated model shown in Figure 5 and modeled by a finite positive value of $L_{OP}$ in Equations (21)–(23) formulated within Nath’s two-ion theory of energy coupling and the torsional mechanism of ATP synthesis successfully explains all the experimental data in the context of mitochondrial energy transduction and OX PHOS.

## 9. Experimental Methods

For the state 3 phosphorylation experiments, rat liver mitochondria were isolated by the method of Schneider and Hogeboom [64] after incorporating the modification by Myers and Slater [65]. Mitochondria containing 1 mg mitochondrial protein/mL were incubated at 25 °C in a vessel with electrodes of oxygen, H^+^, and K^+^. The medium contained 220 mM sucrose, 10 mM Tris succinate, 20 mM Tris HCl, 10 mM Tris Pi, 5 mM MgCl_2_, 1 mM EDTA, 0.4 mM ADP, 1 μg/mL rotenone, and 0.5 μg/mL valinomycin. The final pH was 7.4. In a parallel experiment, instead of ADP, the weak acidic uncoupler 2,4-dinitrophenol was added (3 μM) in order to obtain the same rate of respiration as in the absence of the uncoupler. ADP and ATP were measured by enzymatic assays and Pi was quantitated by a microcolorimetric method as described earlier [27,66].

Aliquots were centrifuged after start of the reaction through silicone into perchloric acid, and the supernatant and acid layer were analyzed for Pi and for K^+^ by an atomic absorption spectrophotometer. Δũ_H_ was calculated by the method of Mitchell and Moyle [67] assuming a mitochondrial matrix volume of 1.8 μL/mg protein and a sucrose-inaccessible space of 3.6 μL/mg protein. The experimental protocols used for studying the state 3 to state 4 transition in mitochondrial oxidative phosphorylation have already been described previously [10].

For the state 4 experiments, mitochondria with ~5 mg protein/mL were incubated in a medium containing 50 mM sucrose, 170 mM mannitol, 1 mM Na-EDTA, 1 mM Tris Pi, 5 mM MgCl_2_, 10 mM Tris succinate, pH 7.3 at 25 °C saturated in oxygen. Additions of 4.5 μg/mL rotenone, 25 μg/mL valinomycin, and varying amounts of uncoupler carbonyl cyanide p-trifluoromethoxyphenylhydrazone, FCCP (0–0.1 nmol/mg protein) or the redox inhibitor sodium malonate (0–20 mM), pH 7.0 were made before addition of Tris succinate. Subsequently, 0.4 mM ADP was added at ~50% saturation. When the oxygen saturation reached a low level of ~10%, a 0.5 mL sample was withdrawn on 0.5 mL of silicone and 0.2 mL of 15% perchloric acid for centrifugation. In the acid layer and supernatant the phosphate and potassium were determined by the colorimetric method and atomic absorption respectively. Δũ_H_ was calculated by assuming equilibration in state 4 of K^+^ and phosphoric acid using the internal volume and sucrose-inaccessible space volume given above. ADP, ATP, and Pi concentrations were determined as above and the state 4 phosphorylation affinity was calculated as described [10,68].

## 10. Concluding Remarks

Starting from the universal concept of entropy production, a wealth of novel thermodynamic, kinetic, and mechanistic insights are provided into the coupling of oxidation and ATP synthesis in the vital biomolecular process of oxidative phosphorylation. The following are the main results and conclusions of this work:

(i) The total rate of free energy dissipation, Φ, in OX PHOS has been evaluated from experimental measurements of thermodynamic fluxes and forces, and the parsing of Φ into its constituent elementary components of ATP synthesis, leak, slip, and other losses has been carried out (Section 2). This information was not available previously.

(ii) The thermodynamic efficiency of energy conversion between oxidation and ATP synthesis, η, has been shown to be obtained from the above information on Φ (Section 3).

(iii) The degree of coupling, q, between oxidation and ATP synthesis has been quantified from the data and shown to be in agreement with calculations made using the formalism of linear nonequilibrium thermodynamics (Section 4, Section 5 and Section 6).

(iv) The optimization of linear biological free energy converters has been explored in considerable detail (Section 7). The state of optimal η has been shown to be compatible with maximum Φ under the imposed constraints of ADP and ATP concentrations and excess Pi, or since generally only extramitochondrial concentrations are measurable, with the ATP/ADP ratio. Contrary to previous biothermodynamic analyses that obtain optimal efficiency, optimal output flux, optimal output power, etc., at different values of the force ratio, $x=A_{P}/A_{O}$ (and are therefore forced to select a particular optimal state), it has been shown by a novel biothermokinetic analysis that all the above optimality functions can be achieved at a single (operating) value of x. This conclusion has been shown to offer novel insights into the nature of coupling between the endergonic and exergonic processes. Such unification could be achieved from molecular structural and functional considerations which reveal that the F_1_F_O_-ATP synthase that catalyzes the chemical reaction is a physically separate complex localized in a different space/compartment (cristae) from the adenine nucleotide translocase (ANT) connecting the inner and outer mitochondrial membranes that is involved in the transport of the ADP substrate and ATP product. However, this separability was not present in the usual biothermodynamic equations, a subtle aspect that has been missed by standard thermodynamic theory. It points to the pressing need to connect the thermodynamics with the structural and functional aspects of the biological system for a proper and complete analysis, something that is only rarely attempted.

(v) In an act-per-act molecular mode of functioning, the true driving force for catalysis by the F_1_F_O_ motor is not the complete thermodynamic function. The concentration-dependent part of the driving force is in fact kinetically associated with a specific transporter, e.g., ANT in the case of OX PHOS.

(vi) New experimental data in state 4 (“static head”) with uncouplers and redox inhibitors of OX PHOS and on respiratory control in the physiological state 3 with ADP and uncouplers has been presented (Section 8 and Figure 6). These experimental observations are shown to be incompatible with Mitchell’s chemiosmotic theory that has been commonly used in the biochemistry textbooks for over 50 years to interpret OX PHOS mechanism and thermodynamics. In view of these and other data, a molecular mechanism that goes beyond the chemiosmotic theory is deemed to be absolutely necessary.

(vii) A novel scheme of coupling based on the previously proposed Nath’s two-ion theory of energy coupling within Nath’s torsional mechanism of energy transduction and ATP synthesis has been shown to pass the test of consistency both with the thermodynamics and the experimental data (Section 8). This theory has been quantified (Equation (28)) and the best-fit values of the coupling coefficients have been regressed from the data. It also provides a molecular interpretation of the transport coefficient, $L_{OP}$, based on succinate binding to the enzyme complexes on the redox side and an attractive explanation of the fundamental phenomenon of respiratory coupling in oxidative phosphorylation that had proved very difficult to explain by other theories of biological energy transduction.

In summary, twenty years since its first proposal, Nath’s torsional mechanism of energy transduction and ATP synthesis has been shown to take us beyond the chemiosmotic theory. The alternative theory is now perfectly poised to catalyze the progress of experimental and theoretical research in this important interdisciplinary field. It is hoped that a large number of workers will adopt the theory in the further design of experimental and computational work and for interpretation of their results, and that it will rapidly become the standard theory in the field. Textbooks in the biological sciences and allied fields now have the exciting challenge and opportunity, and the author would like to add, responsibility, to communicate the new developments in the subject and thereby catalyze this process of dissemination of scientific knowledge. It would be interesting to be witness to this enterprising endeavor.

## Figures and Tables

**Table 1:** Theoretical thermodynamic efficiencies, η, (in %) for single and two-variable optimization by biological free energy transducers.

Type of Optimization	Maximum Output Flow, J_P_	Maximum Output Power, J_P_A_P_	Maximum Economic Output Flow, J_P_η	Maximum Economic Output Power, J_P_A_P_η
Single variable with q = 1	<0	50	50	67
Two variable [13,59]	23.6	41.4	53.5	61.8

**Table 2:** The degree of coupling, q, in optimal states for which the tabulated output function is maximal at optimum efficiency of energy coupling, η, for various mechanisms of uncoupling.

Output Function	Normalized (As in Stucki [13])	Leak	Redox Slip	ATPase Slip
		(Without Normalization [9,10,11,57,59])		
J_P_	0.786	0.612	0.100	0.289
J_P_A_P_	0.909	0.882	0.864	0.886
J_P_η	0.953	0.946	0.941	0.944
J_P_A_P_η	0.973	0.969	0.969	0.969

**Table 3:** Thermodynamic fluxes and forces corresponding to the same respiratory rate in the presence and absence of 2,4-dinitrophenol for rat liver mitochondria with succinate as substrate.

2,4-dinitrophenol (μM)	J_O_(natom O/(mg min))	J_P_(nmol ATP/(mg min))	Δũ_H_ (mV)
0	98	152	233 ± 5
3	97	105	200 ± 5
